# Edgewise Compressive Behavior of Composite Structural Insulated Panels with Magnesium Oxide Board Facings

**DOI:** 10.3390/ma14113030

**Published:** 2021-06-02

**Authors:** Łukasz Smakosz, Ireneusz Kreja, Zbigniew Pozorski

**Affiliations:** 1 Department of Structural Mechanics, Faculty of Civil and Environmental Engineering, Gdańsk University of Technology, ul. Gabriela Narutowicza 11/12, 80-233 Gdańsk, Poland; ireneusz.kreja@pg.edu.pl; 2 Institute of Structural Analysis, Faculty of Civil and Transport Engineering, Poznan University of Technology, ul. Piotrowo 5, 60-965 Poznań, Poland; zbigniew.pozorski@put.poznan.pl

**Keywords:** composites, sandwich panel, composite structural insulated panel, magnesium oxide board, bimodular material, experimental mechanics, computational mechanics, finite element analysis

## Abstract

Edgewise compression response of a composite structural insulated panel (CSIP) with magnesium oxide board facings was investigated. The discussed CSIP is a novel multifunctional sandwich panel introduced to the housing industry as a part of the wall, floor, and roof assemblies. The study aims to propose a computational tool for reliable prediction of failure modes of CSIPs subjected to concentric and eccentric axial loads. An advanced numerical model was proposed that includes geometrical and material nonlinearity as well as incorporates the material bimodularity effect to achieve accurate and versatile failure mode prediction capability. Laboratory tests on small-scale CSIP samples of three different slenderness ratios and full-scale panels loaded with three different eccentricity values were carried out, and the test data were compared with numerical results for validation. The finite element (FE) model successfully captured CSIP’s inelastic response in uniaxial compression and when flexural action was introduced by eccentric loads or buckling and predicted all failure modes correctly. The comprehensive validation showed that the proposed approach could be considered a robust and versatile aid in CSIP design.

## 1. Introduction

The composite structural insulated panel (CSIP) is a novel product introduced to the housing industry as a part of the wall, floor, and roof assemblies in low-rise buildings. It is a type of multifunctional sandwich panel that combines enveloping, thermoinsulational, and structural roles. Composite materials with low weight to strength ratio and modularized components allow to significantly reduce the time and cost of transport and assembly, making them an attractive alternative to traditional construction materials [1,2,3,4,5]. The CSIP is a developed version of a structural insulated panel (SIP), which uses mainly wood-based facing materials, such as oriented strand board (OSB), that are prone to biological and environmental degradation [6,7]. The use of the right composite facings can solve this problem and, depending on the type of material used, introduce additional advantages.

**Figure 1:**
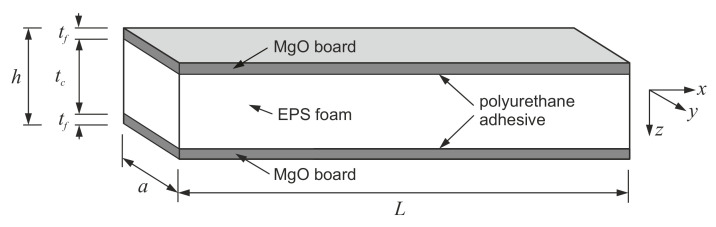
Schematic layout of the analyzed CSIP.

The subject of the present research is a CSIP with magnesium oxide board (MgO board) facings and an expanded polystyrene (EPS) core, bound together by a polyurethane adhesive (Figure 1). The MgO board is a relatively new cladding material, composed of a magnesia cement mortar matrix and a glass-fiber mesh reinforcement. Such use of the MgO board provides the panel with high strength and stiffness, immunity to biological corrosion, flame retardancy, and environmental sustainability [8,9,10,11]. The analyzed CSIP overcomes the disadvantages of a traditional SIP and allows to create more durable and eco-friendly buildings.

The CSIP under consideration is intended for use as a structural element of walls, which means it has to be suitable for in-plane load transfer. The need for a sufficient thermal insulation and structural strength results in a relatively high total thickness to length ratio. This type of geometry makes it prone to premature initiation of local damage, and the actual failure pattern is difficult to predict at the design stage. Possible failure modes, when subjected to in-plane compression, include yielding of facings, core shear, global buckling, inward local buckling (core crushing), and outward local buckling (delamination) [12]. The prominent difference in facing and core stiffness and the complex nature of their interactions make a prediction of CSIP failure mode a challenging task. Therefore, it is essential to provide a computational tool well-suited for this type of composite.

Several investigations on the subject of sandwich panel behavior under axial loads were carried out in recent years. The compressive behavior of sandwich column samples with carbon/epoxy facings and two types of core material, polyvinyl chloride foam and aluminum honeycomb, was investigated in [13]. Local buckling failure was observed in the soft foam core samples, whereas no wrinkling occurred in the honeycomb sample, due to its high thickness–direction stiffness. Fundamental analytical expressions allowed to predict the wrinkling load when it appeared before the core yield, but the formulas had to be modified to account for the stiffness loss in cases where the core failed first. CSIPs with thermoplastic glass/polypropylene facings and an EPS core were analyzed in [14,15]. The first report [14] concentrates on global buckling failure of small-scale samples caused by concentric and eccentric loads. The authors derive formulae that consider the orthotropic facings and the core shear deformation to predict the elastic buckling load successfully. The second study [15] concerns full-scale CSIPs subjected to eccentric compression. All tested panels failed by local buckling, and an analytical model for critical wrinkling stress was proposed and validated for the elastic range. Furthermore, a 3D continuum FE model with geometric nonlinearity was used for a parametric study, highlighting the possibilities of such an approach in CSIP design. Mechanical behavior of panels with low density polyethylene facings and a lightweight polyethylene foam core under edgewise compression was investigated in [16]. An experimental study on small-scale samples was carried out, utilizing strain and out-of-plain deformation measurements, and multiple cases of localized buckling were captured. A high fidelity 3D continuum FE model accounting for thickness irregularities as well as material and geometrical nonlinearities was created and successfully validated against experimental results. Afterward, the model was used in a parametric study to assess the sensitivity of panel’s response to changes in layers’ thickness and interface irregularities. An extensive study on the influence of slenderness ratio on the compressive behavior of glass fiber reinforced polymer (GFRP) facings and polyurethane foam core sandwich panels’ compressive response was carried out in [17]. The observed failure types were global buckling, wrinkling, GFRP yield, and core shear. The authors correlated the failure modes to the slenderness ratio and proposed analytical expressions for ultimate load prediction in the elastic range. An investigation of axial performance of single sandwich wall panels and panel assemblies jointed with a novel connector system was performed in [18]. The observed failure modes were local buckling of GFRP skins, and global instability resulting from delamination between the core and facings. A linear elastic theoretical study was carried out and the obtained results were in a reasonable agreement with the experimental data. Load-bearing sandwich panels with glass fiber reinforced polymer (GFRP) facings and a foam-web core subjected to edgewise compression were discussed in [19]. Outward local buckling and compressive facing yield failure types were observed, depending on the foam-web layout. Analytical formulae calculating critical local buckling stress, and axial load capacity of the panel were derived and validated in an experimental examination. Elastic range consideration was sufficient in this case as well. One of the few examples of MgO board strength research was presented in [9]. The structural behavior of wall-panels with MgO board facings and a glass fiber reinforced polyurethane foam core was investigated. Full-scale panels with different support assemblies and a panel with a damaged facing were tested in uniaxial compression. The observed failure modes were facing buckling followed by vertical cracking and facing cracking due to shear sliding. It was also observed that the presence of local damage in the board caused cracks propagating from the weakened area and led to a significant reduction of the load-bearing capacity of the panel. Examples of nonlinear FE approach to sandwich panel analysis can be found in [20,21]. Numerical studies of composite response to local loads were performed with consideration of both material and geometrical nonlinearities. Core layers were modeled with continuum solids and facings were treated as structural shells. This approach allowed for a more detailed insight into sandwich layer interactions. A high fidelity method of sandwich panel FE modeling is presented in [22]. Nonlinear material behavior was applied to all components, taking into account the difference in tensile and compressive responses of textile-reinforced cement faces, and high deformability of an extruded polystyrene foam (XPS) core in compression. The numerical approach was validated by comparison of surface strain results in different layers with comprehensive digital image correlation data. Structural behavior of SIPs with OSB facings subjected to concentric and eccentric compression was analyzed in a joint experimental and analytical study in [23]. Full-scale panels with different configurations of slenderness ratio and foam core type were tested, and the observed force-deflection responses were predominately linear until failure. Failure modes consisted of facing crushing at different locations, core shear, core rupture near the interface, and debonding at the adhesive layer. Several design recommendations, along with empirical expressions for SIP’s ultimate axial strength, were proposed.

The analyzed CSIP and its components were subjected to various mechanical tests to identify its failure patterns and establish material properties of the MgO board and the EPS core [24,25]. Both core and facing yield were noted before failure initiation, therefore limiting the computational model to the elastic range would be inadequate. Material and geometrical nonlinearities had to be considered. Moreover, it was observed that the structural response and the parameter values depended strongly on the stress state of the materials and that the most notable differences occurred between compression and tension. The observed material bimodularity was incorporated into a preliminary FE model, which significantly improved the simulation results’ overall quality. An attempt to use this approach for CSIP edgewise compression analysis was made [26]. The numerical analysis produced qualitatively acceptable results; however, the samples’ stiffness and strength were considerably underestimated. Quite recently, a refined description of the bimodular material model was proposed and positively validated [27]. As a result, a notable improvement of similarity between numerical and experimental curves, and accuracy of failure mode prediction was achieved for flexural behavior.

In the current work, the refined bimodular FE approach was used to simulate the behavior of the MgO board CSIP under concentric and eccentric edgewise compression. The validation of the numerical model was accomplished by comparing its outcomes with the results of laboratory tests performed on samples of different geometries and eccentricity values. Both small-scale and full-scale samples were investigated for comprehensive validation. The study aims to propose a robust, versatile computational framework that can be used as a reliable design aid for predicting CSIP failure modes in compression.

## 2. Materials and Methods

### 2.1. Experimental Analysis

A series of laboratory edgewise compression tests was executed on a variety of CSIP samples (Table 1). Small-scale CSIP columns of three different heights (L1, L2, L3) were tested under uniaxial compression, and full-scale panels were subjected to compression with three different eccentricity values (e0, e1, e2). The test series aimed to produce a variety of compressive responses and failure modes to provide experimental data for the comprehensive validation of the FE model.

**Figure 2:**
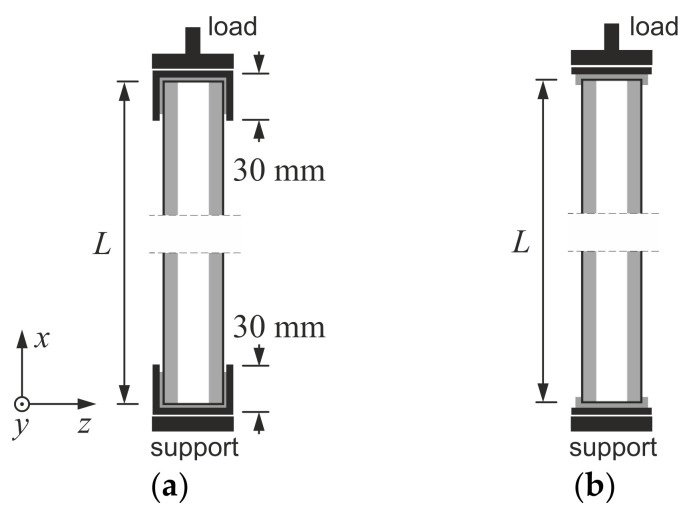
Schematic diagram of small-scale laboratory tests: (**a**) L1 and L2, (**b**) L3.

Small-scale edgewise compression tests were performed based on the procedure given in [28]. The tests were conducted on an Instron 5569 machine (Instron, Buckinghamshire, UK) using displacement control and a continuous recording of cross-head movement, *u_x_*, and reaction force, *F_x_*. CSIP columns’ dimensions were assumed with a gradually increasing slenderness in an attempt to produce both facing yield and global buckling failure modes. Since the original panel was too thick to observe buckling behavior in small-scale, all specimens were modified by removing the central portion of the core and using an adhesive to create columns of reduced thickness. This interference in the composite layout did not influence the compressive behavior of the samples in any noticeable way. It was noted that the EPS cores of the source panels had two different densities: 15 kg/m^3^ (EPS15) and 21 kg/m^3^ (EPS21). Other than that, the cross-section of all samples remained constant, and three different heights were considered (Table 1). In the case of L1 and L2 columns, support profiles with 30 mm high flanges were used (Figure 2a). The flanges were discarded for the L3 column to reduce rotational stiffness and increase slenderness (Figure 2b). A stabilizing layer of mortar was applied in all cases to ensure uniform stress distribution.

**Figure 3:**
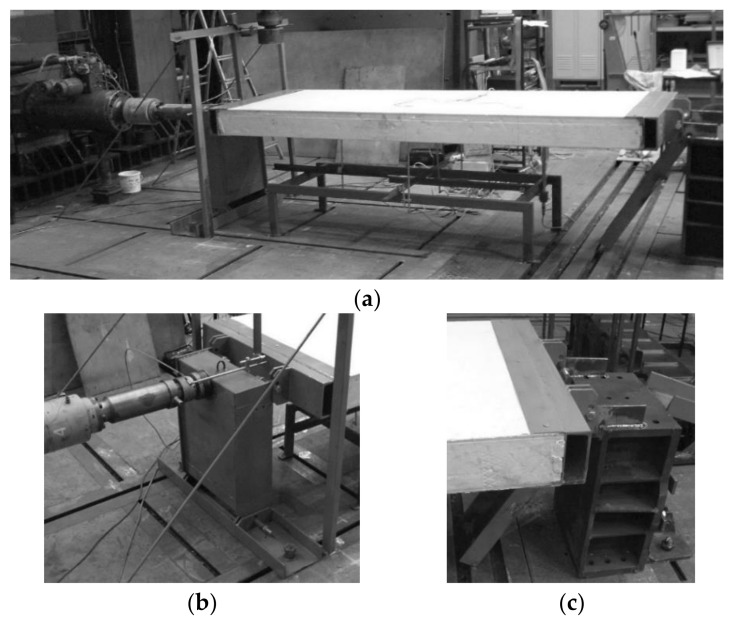
Full-scale CSIP test stand: (**a**) overall view, (**b**) loading assembly, (**c**) support assembly [26].

**Figure 4:**
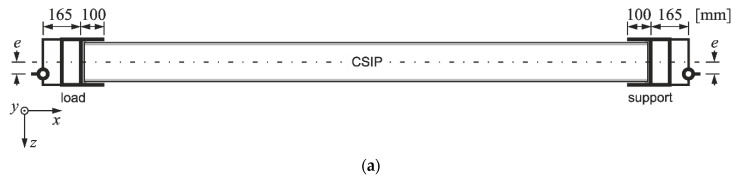

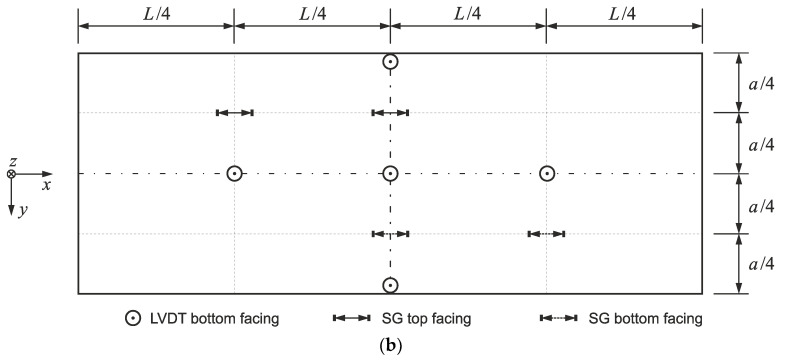
Schematic diagram of full-scale laboratory tests: (**a**) load and support, (**b**) positions of measuring devices.

A full-scale CSIP compression test procedure was developed based on small-scale research and panel application guidelines provided by the producer. The test stand comprised an Instron Labtronic^®^ 8800 structural testing system (Instron, Buckinghamshire, UK) with a NBC Elettronica TA10 load cell (N.B.C. Elettronica Group s.r.l., Delebio, Italy) and a tested panel (LS Tech-Homes S.A., Czechowice-Dziedzice, Poland) mounted horizontally in two steel profiles acting as pin supports (Figure 3). The mounting profiles were designed to warrant sufficient rigidity with 165 mm distance from a panel’s edge to the pin, and a 100 mm high flange (Figure 4a). A stabilizing layer was used between the sample and the profiles for uniform stress distribution. The assembly was attached to a steel frame, that allowed for a horizontal movement of the loading profile (Figure 3b) and blocked all translations of the support profile (Figure 3c). The connection between the pin supports and the steel frame allowed to apply loads with a set eccentricity value. Three levels of eccentricity were selected to produce a substantially varied response: 0, *d*/6 (27 mm) and *d*/3 (54 mm), where *d* is distance between facing centroids, *d* = *h* − *t_f_*. All tests were performed under displacement control with continuous recording of reaction force, *F_x_*, horizontal displacement, *u_x_*, using cross-head movement and a linear variable differential transformer (LVDT), vertical displacements, *u_z_*, using LVDTs, and facing longitudinal strains, *ε_x,f_*, using strain gauges (SG). The measuring devices were LVDTs with a precision of 0.01 mm, and tubular strain gauges with a grid length of 60 mm. Measuring devices’ placement is shown in Figure 4b.

Information considering the number of tested samples, the EPS core’s density, specimen dimensions, and characteristics affecting slenderness is summarized in Table 1. The slenderness ratio was calculated from:

(1) $$\lambda =L_{e}\sqrt{\frac{A_{f}}{J_{y}}}=L_{e}\sqrt{\frac{2t_{f}a}{\frac{a}{12}\left(h^{3}-t_{c}{}^{3}\right)}}=L_{e}\sqrt{\frac{24t_{f}}{h^{3}-t_{c}{}^{3}}},$$

where: *L_e_*—effective length; *A_f_*—cross-sectional area of facings; *J_y_*—moment of inertia of facings; remaining symbols in accordance with Figure 1. The effective length was assumed as: *L_e_* = *L*/2 for specimens with rotational constraints (L1, L2), *L_e_* = *L* for the L3 sample, and *L_e_* = *L* + 2 × 165 mm to account for mounting profiles’ height for full-scale panels (e0, e1, e2).

### 2.2. Numerical Analysis

The proposed approach was applied to perform a numerical study, validate the FE model, and assess its viability as a design aid tool. Both small-scale and full-scale tests described in Section 2.1 were reproduced as simulations and the computational results were compared with the test data. In total, six numerical test assemblies were created using ABAQUS software [29] (version 6.11, Dassault Systèmes, Providence, RI, USA). The computations were supplemented by an author’s procedure, implemented to account for dependence of material response from stress state [27].

**Figure 5:**
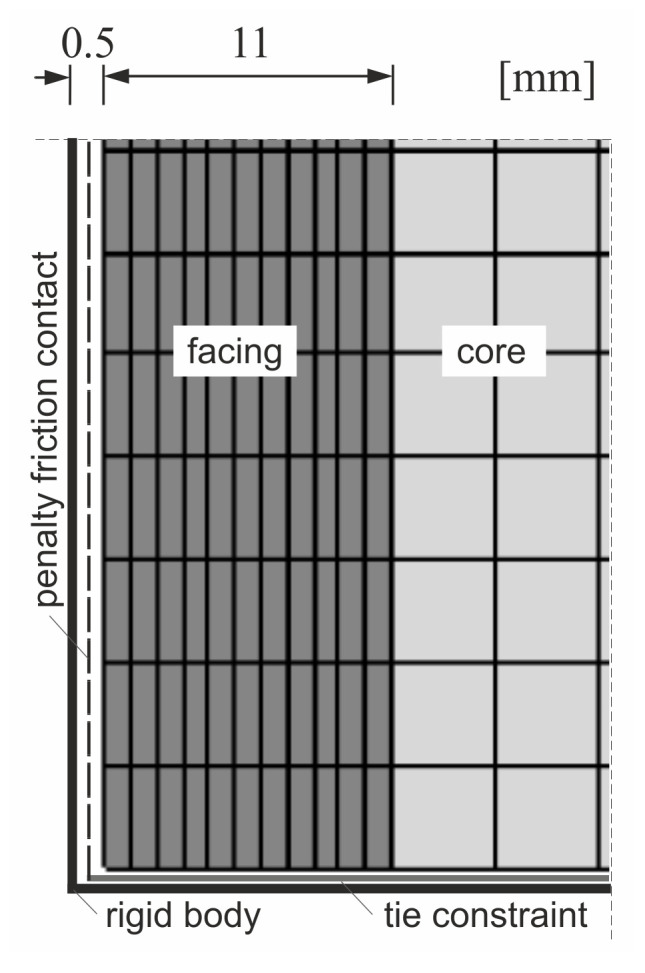
FE mesh section in the support area and rigid body contact interactions.

A continuum approach was taken and all simulations were performed in plane stress state. The test samples were discretized using four-node elements with reduced integration and hourglass control. A regular geometry mesh established in a convergence study, consisting of 4 mm × 4 mm elements in the core area and 1 mm × 4 mm in the facings, was used in all cases (Figure 5). Sample dimensions and layer arrangements were adopted in accordance with Table 1. Perfect bonding was assumed between facings and core constituents since no pre-failure delamination was observed in laboratory tests.

**Figure 6:**
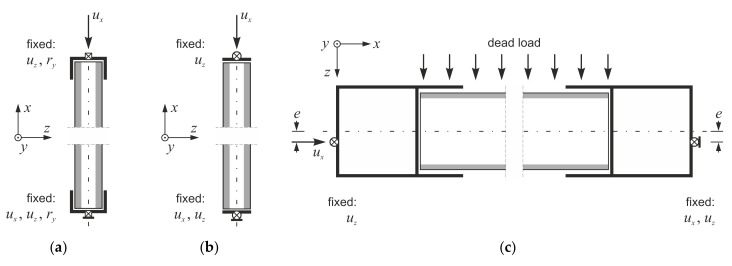
Boundary conditions used in simulations of (**a**) L1, L2, (**b**) L3, (**c**) e0, e1, e2 tests.

The loading and support profiles were idealized as linear rigid bodies to ensure indirect load transfer and uniform stress distribution in the analyzed samples. Two types of interactions between a specimen and a rigid body profile were defined: (1) tie constraint at the edge perpendicular to the direction of compression and (2) penalty friction with a 0.1 coefficient on the sides parallel to the direction of compression (Figure 5). Gaps of 0.5 mm between a modeled sample and a rigid profile were created on the edges with the frictional contact to reflect small clearances that were present in laboratory tests. The boundary conditions and loads were prescribed on the rigid profiles’ reference points (Figure 6). The profiles’ geometries were adjusted to match the experimental support conditions: flanges were used for L1 and L2 specimens (Figure 6a), no flanges were created for the L3 column (Figure 6b), and simplified shapes were generated for full-scale panels (Figure 6c).

The computations were realized as geometrically nonlinear static analysis. Samples were loaded using displacement control in all simulations (Figure 6). In case of full-scale tests dead load was considered additionally, due to horizontal orientation of the specimens (Figure 6c), with mass densities of 1130 kg/m^3^ for MgO board and 21 kg/m^3^ for EPS [24,25]. Both core and facing constituents were defined with isotropic elastic–plastic material models. An extended Drucker–Prager model with hyperbolic yield criterion, available in the ABAQUS software [29], was applied for both constituents. MgO board property values were characterized by a substantial scatter [24,25] so instead of using averaged values two descriptions representing experimental result boundaries were defined as MgO min and MgO max. A damage initiation criterion defined in ABAQUS [29] in terms of equivalent plastic fracture strain, *ε^pl,eq^*, and stress triaxiality factor, *η*, was used for failure mode prediction. Parameter values defining the material model are presented in Table 2.

Computations were terminated when the damage initiation criterion variable (DICV) reached unity. All experimental samples lost their load-bearing capacity after initial failure, so reaching the criterion fulfillment was sufficient to identify the failure mode, and damage evolution analysis was not performed. A stabilization algorithm with numerical damping factor of 1 × 10^−9^ was used to prevent convergence issues occurring directly before failure.

An author’s procedure was supplemented during computations to account for the material bimodularity effect. The procedure allowed to prescribe material property values in all integration points, depending on their stress states at the beginning of each increment in an automated manner. The algorithm generates a stress state variable (SSV) based on a following set of conditions:

(2) $$\mathrm{SSV}=\left\{\begin{array}{l} -1\;\;\;\;\;\;\;\;\;\;\;\;\;\;\\ \left|\sigma_{\max}/\sigma_{\min}\right|-1\\ 0\;\;\;\;\;\;\;\;\;\;\\ \sigma_{\min}/\sigma_{\max}+1\\ 1\;\;\;\;\;\;\;\;\;\; \end{array}\right.\;\;\;\;\begin{array}{l} \mathrm{when}\;\sigma_{\max}\leq 0\;\;\;\;\;\;\\ \mathrm{when}\;\left|\sigma_{\min}\right|>\left|\sigma_{\max}\right|\\ \mathrm{when}\;\left|\sigma_{\min}\right|=\left|\sigma_{\max}\right|\\ \mathrm{when}\;\left|\sigma_{\min}\right|<\left|\sigma_{\max}\right|\\ \mathrm{when}\;\sigma_{\min}\geq 0\;\;\;\;\;\;\; \end{array},$$

where *σ*_min_ = min(*σ*_1_, *σ*_2_, *σ*_3_), *σ*_max_ = max(*σ*_1_, *σ*_2_, *σ*_3_), and *σ*_1_, *σ*_2_, *σ*_3_ are the principal stress values. SSV generated from (2) describes stress state in any given integration point and can be used with most material models as a field variable, enabling definition of multiple values for a selected parameter. Characteristic states for which parameter values were defined in this finite element analysis (FEA) were SSV = −1 (compression), SSV = 0 (shear), and SSV = 1 (tension). In cases where SSV values fell between the defined characteristic states, parameter values were automatically obtained through linear interpolation. A summary of characteristic SSV values and corresponding material parameter values used in the analysis is shown in Table 2. The majority of presented data were established in course of an experimental investigation, supplemented by a literature study and a parameter identification analysis as an extensive part of previous research [25,26,27].

## 3. Results

Experimental data obtained from small- and full-scale compression tests are presented and compared with computational results obtained from the proposed FE model. Four types of results are discussed: (1) SSV distribution maps at failure initiation (only in FEA), (2) failure modes, (3) force–displacement curves and (only in full-scale) force–strain curves, (4) failure stress values.

### 3.1. Small-Scale Sample Tests

**Figure 7:**
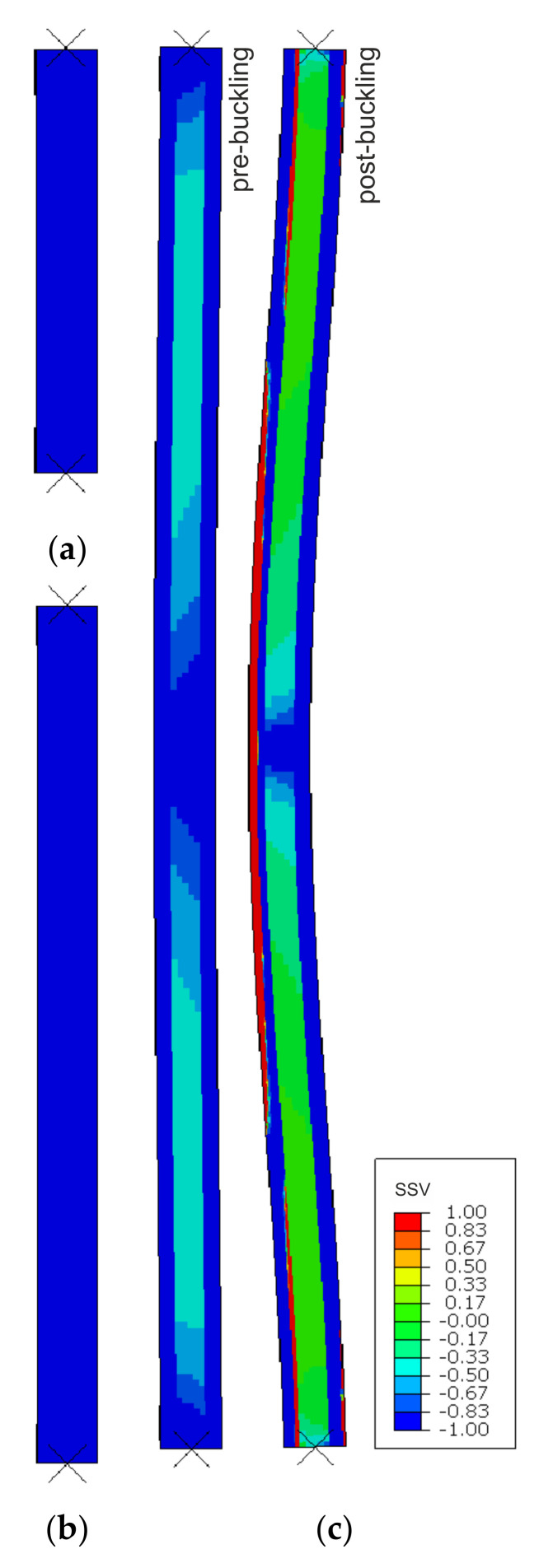
SSV distribution in compression simulations of (**a**) L1, (**b**) L2, and (**c**) L3 samples.

SSV distribution maps are presented in Figure 7. Only the MgO min variant is shown as MgO max outcomes are very similar. Both shorter samples, L1 and L2, were identified as wholly under compression (Figure 7a,b), whereas the highest column, L3, was recognized as under compression before buckling and shifted into a flexural deformation when the buckling occurred (Figure 7c). After the critical load was reached and further vertical displacement was applied, one facing remained nearly entirely under compression, and in the other substantial areas under tension appeared in the center and near the supports. All of the SSV maps depict physically reasonable behavior and exemplify that the author’s procedure works as intended.

**Figure 8:**
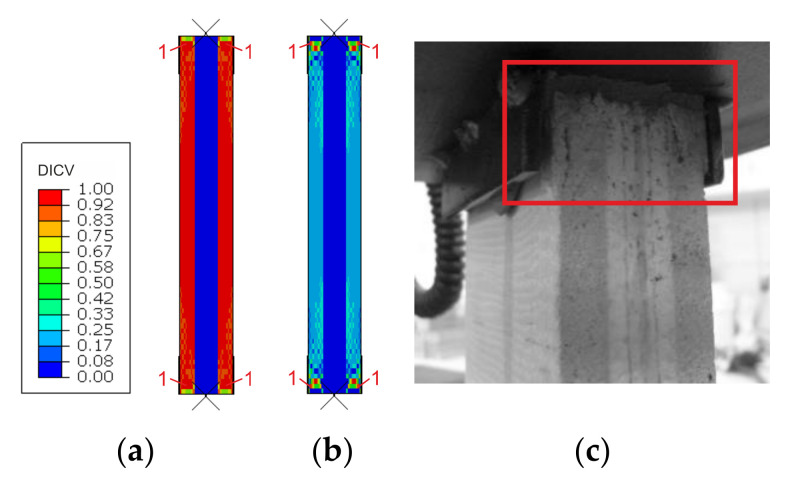
Comparison of failure modes in the L1 sample compression test obtained from FEA (**a**) MgO min, (**b**) MgO max variants, and (**c**) experimental observation.

**Figure 9:**
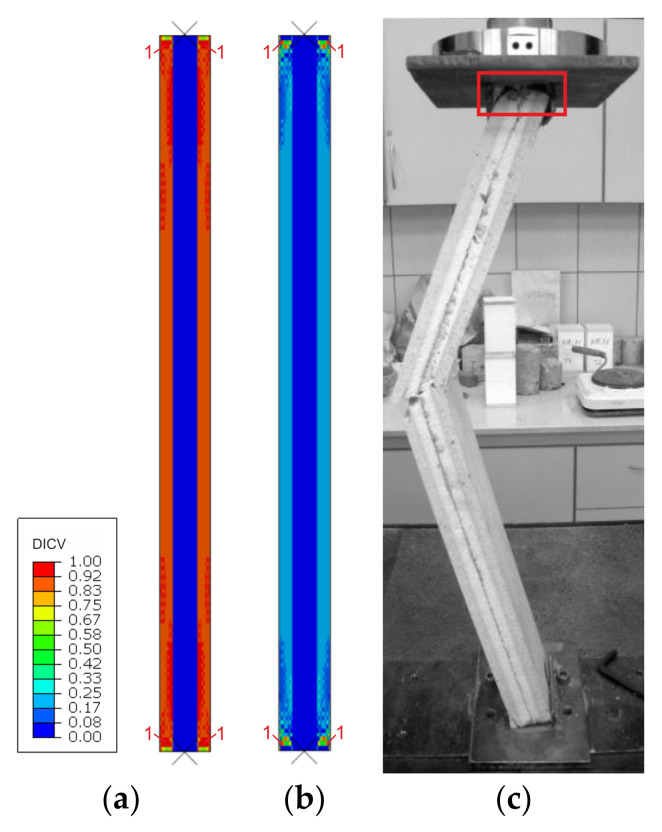
Comparison of failure modes in the L2 sample compression test obtained from FEA (**a**) MgO min, (**b**) MgO max variants, and (**c**) experimental observation.

**Figure 10:**
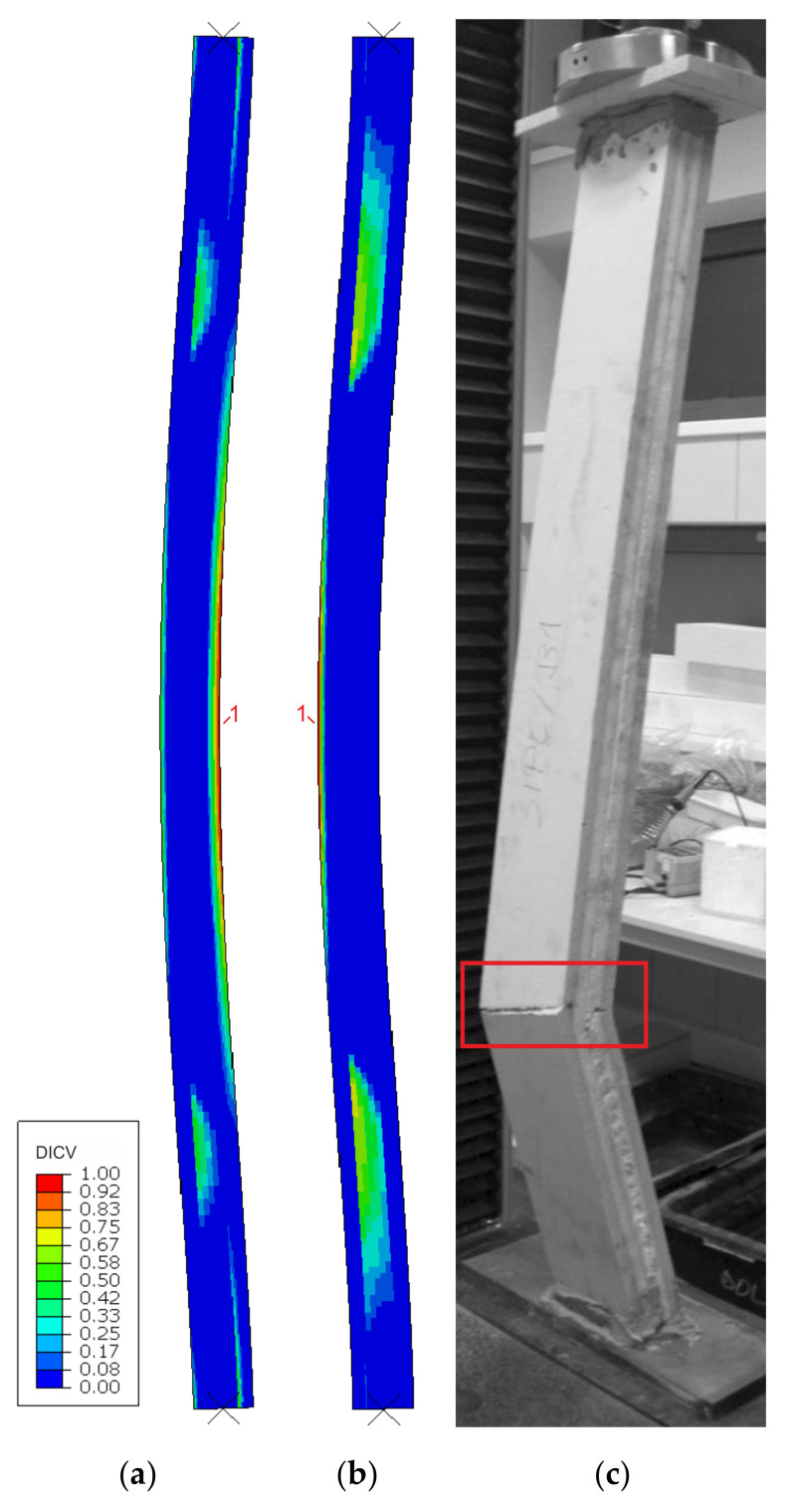
Comparison of failure modes in the L3 sample compression test obtained from FEA (**a**) MgO min, (**b**) MgO max variants, and (**c**) experimental observation.

Failure modes are presented as experimental observations and DICV distributions in Figure 8, Figure 9 and Figure 10. In both shorter samples, L1 and L2, the failure initiated on the edges of the facings, in the contact zone with the support profiles, and in both cases, the computational predictions agree with laboratory test observations (Figure 8 and Figure 9). No flexural deformation occurred in the L1 sample throughout the experiment, neither in laboratory specimens nor in their numerical representation. A post-failure deflection occurred in the L2 sample laboratory test (Figure 9c), but since the FEA’s focus was on the failure initiation, this behavior was not investigated further in simulations (Figure 9a,b). The use of the MgO min and MgO max variants did not affect the location of failure initiation points, however, for MgO min, both facings were recognized as under significant strain with DICV values close to 1 across the whole area (Figure 8a and Figure 9a), whereas for MgO max, the peak DICV values appeared only in concentrated areas near the supports (Figure 8b and Figure 9b). A global buckling occurred in the highest column, L3, and failure initiated in its central section (Figure 10). It can be seen that the numerical sample deformed symmetrically (Figure 10a,b), whereas the physical specimen cracked around one-third of its height (Figure 10c). The imperfection of support conditions and sample positioning in the laboratory test was the most probable cause of this difference.

**Figure 11:**
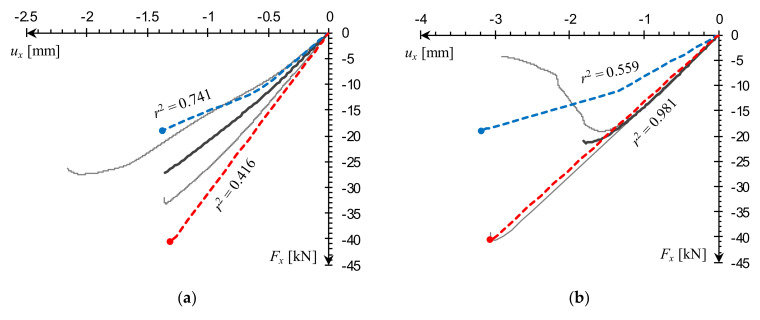

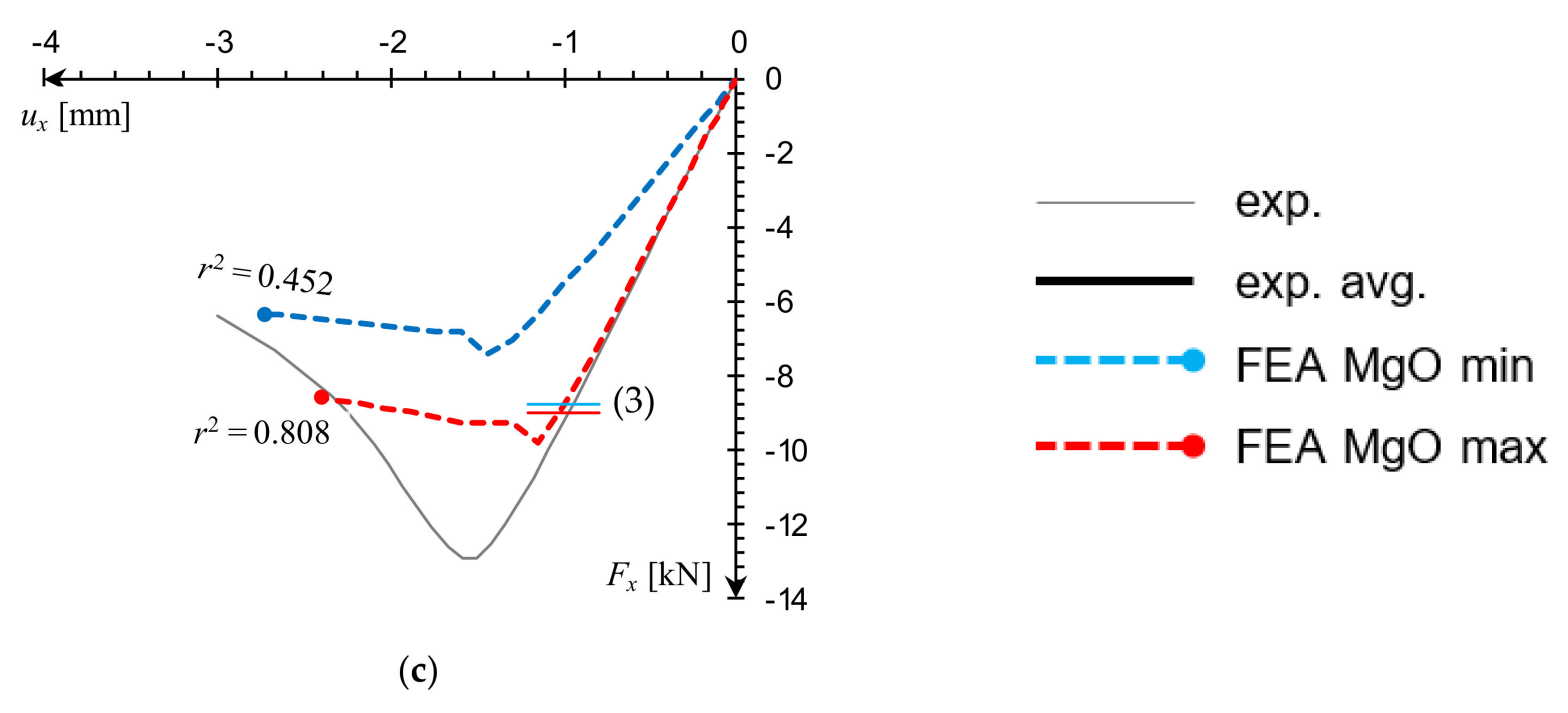
Comparison of small-scale sample compression *F_x_*(*u_x_*) curves obtained from FEA against experimental data for (**a**) L1, (**b**) L2, (**c**) L3.

Force–displacement, *F_x_*(*u_x_*), experimental curves for individual samples, averaged when more than one reading was available, are compared with FE model outcomes for MgO min and MgO max variants in Figure 11. Every computational curve was matched against a corresponding experimental curve by resampling the analyzed datasets in their shared domain and calculating a coefficient of determination, *r*^2^, used here as a measure of curve similarity [30]. The closer the *r*^2^ value is to unity, the stronger the resemblance of the computational curve to the experimental one.

Examination of the L1 sample results shows that the material model variant outcomes encompass the experimental series quite well. The values of *r*^2^, obtained in relation to the averaged curve, range from 0.4 to 0.7 and similarity with individual laboratory specimens is even more pronounced (Figure 11a). The L2 sample FEA plot for the MgO max variant is in very good agreement with the averaged experimental data (*r*^2^ nearing unity) and a nearly exact match with one of the individual specimen results (Figure 11b). In the MgO min case, the plot shape diverges from experimental curves, but the predicted failure load is in a satisfactory agreement with the minimal laboratory reading.

For the L3 column, the MgO max curve shape is very similar to the experimental plot, while the MgO min prediction is visibly underestimated (Figure 11c). It is worth to note a qualitative difference between plotlines recorded in global buckling, and the ones corresponding to failure by facing edges cracking. In the first case there is a smooth transition from the peak and into the post-critical slope (Figure 11c), while in the latter irregular drops are visible (Figure 11a,b). An equivalent buckling load was additionally estimated using a formula for sandwich columns with core shear effect, derived in [14] and adjusted to assume facing material isotropy:

(3) $$F_{x}{}^{{}_{eq}}=\frac{F_{E}}{1+\frac{F_{E}}{A_{s}G_{c}}}=\frac{\pi^{2}}{L_{e}{}^{2}}\frac{E_{f}J_{y}}{\left(1+\frac{\pi^{2}}{L_{e}{}^{2}}\frac{E_{f}J_{y}}{A_{s}G_{c}}\right)},$$

where: *F_E_*—critical buckling load; *A_s_* = *a*(*h* + *t_c_*)/2—shear area of the column; *G_c_*—core shear modulus; *L_e_*—effective length; *E_f_*—modulus of elasticity of facings; *J_y_* = *a*(*h*^3^ − *t_c_*^3^)/12—moment of inertia of facings about the centroid of the panel. The L3 sample buckling load obtained for parameters listed in Table 2, ranges from 8.8 to 9.1 kN which fits within the numerical prediction (Figure 11c). Computational and analytical results are both significantly lower than the laboratory test reading. Again, this can be explained by the influence of boundary conditions. In both FEA and analytical estimation (3), a free rotation was assumed on both ends, whereas laboratory sample supports had some rotational stiffness.

The result summary is shown in Table 3, with experimental failure stress obtained from (4) assuming *e* = 0.

(4) $$\sigma_{x,f}=\frac{F_{x}}{2at_{f}}+\frac{F_{x}e}{\frac{a}{12}\left(h^{3}-t_{c}{}^{3}\right)}\frac{h}{2},$$

### 3.2. Full-Scale CSIP Tests

**Figure 12:**
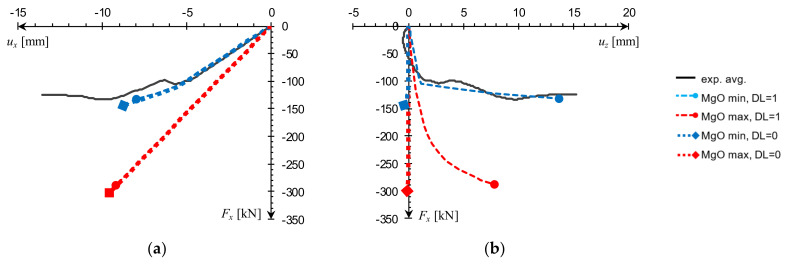
Dead load influence on e0 panel FEA results: (**a**) *F_x_*(*u_x_*), (**b**) *F_x_*(*u_z_*) at L/2.

Dead load influence was additionally considered in the full-scale FEA since the compressed panels were oriented horizontally. Due to CSIPs’ low weight, the obtained mid-span vertical deflection was less than 0.9 mm; however, it did play a notable role in the case of concentric compression test simulation. A comparison of numerical results obtained with and without dead load consideration in relation to experimental data is shown in Figure 12. It can be seen that while its influence on horizontal deflection was insignificant (Figure 12a), it caused a qualitative change in the nature of vertical deflection response (Figure 12b).

**Figure 13:**
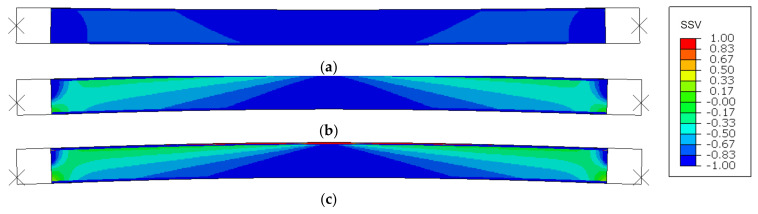
SSV distribution in natural-scale panel compression simulations for (**a**) e0, (**b**) e1, (**c**) e2.

The SSV distribution maps at failure initiation are presented in Figure 13. The facings of the e0 sample (*e* = 0) were identified as being entirely under compression, while substantial portions of the core edged towards shear (Figure 13a). The slight downward deflection of the panel is caused by the consideration of the dead load. The deflection of two remaining CSIPs is directed upwards, due the compressive load placement. There is a noticeable flexural deformation in the e1 sample (*e* = 27 mm). Both facings remain in the state of compression, but large portions of the core are recognized as approaching shear (Figure 13b). In the e2 sample (*e* = 54 mm), the flexural deformation is more pronounced (Figure 13c). The whole bottom facing is identified as being under compression; however, tension dominates in the central part of the top facing. Portions of the core that are not under uniaxial compression continue to grow and translate into small areas staying under pure shear. The shear stress state progression in the core coincides with changes in each specimen’s vertical deflection direction and intensity. It appears to be a consequence of flexural action becoming more pronounced as the eccentricity value increases. The presented results indicate a physically reasonable pattern of dependency between eccentricity value and stress state distribution in the core and facings.

**Figure 14:**
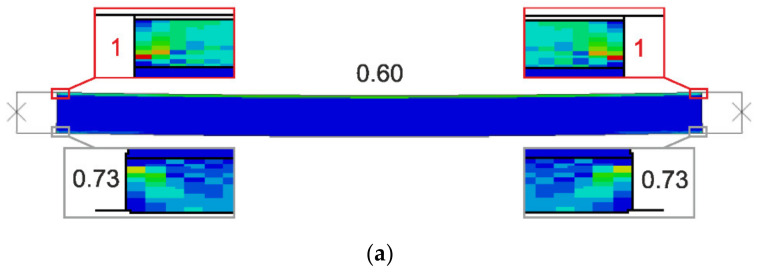

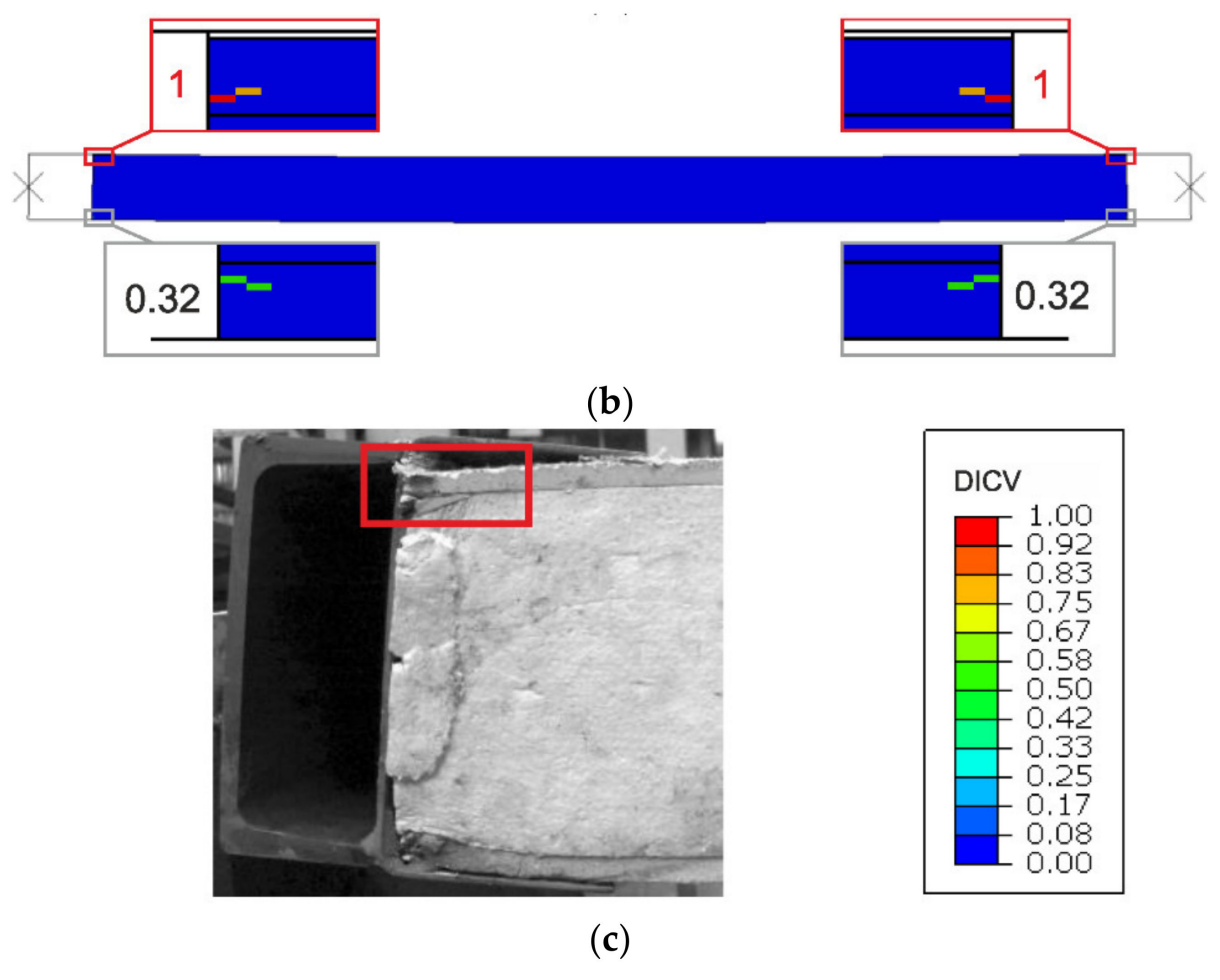
Comparison of failure modes in natural-scale e0 panel compression test; FEA results: (**a**) MgO min, (**b**) MgO max; (**c**) experimental observation.

**Figure 15:**
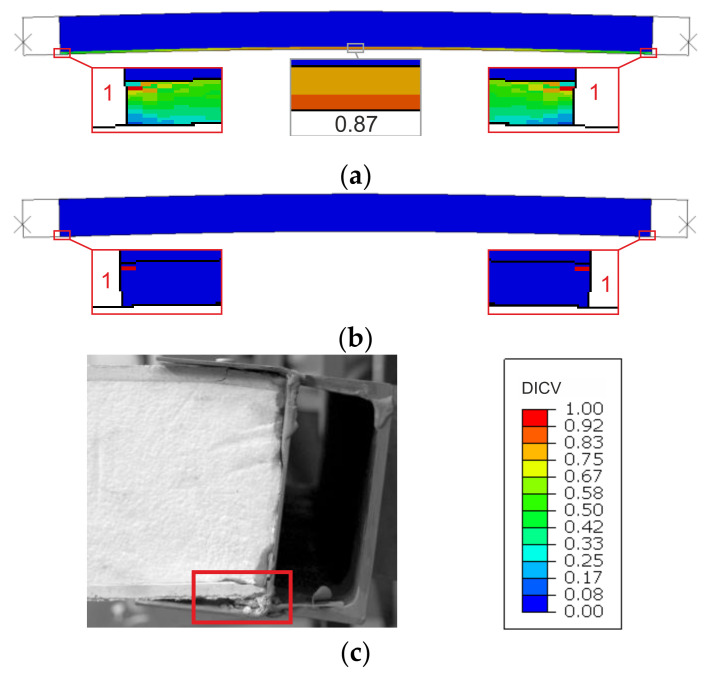
Comparison of failure modes in natural-scale e1 panel compression test; FEA results: (**a**) MgO min, (**b**) MgO max; (**c**) experimental observation.

**Figure 16:**
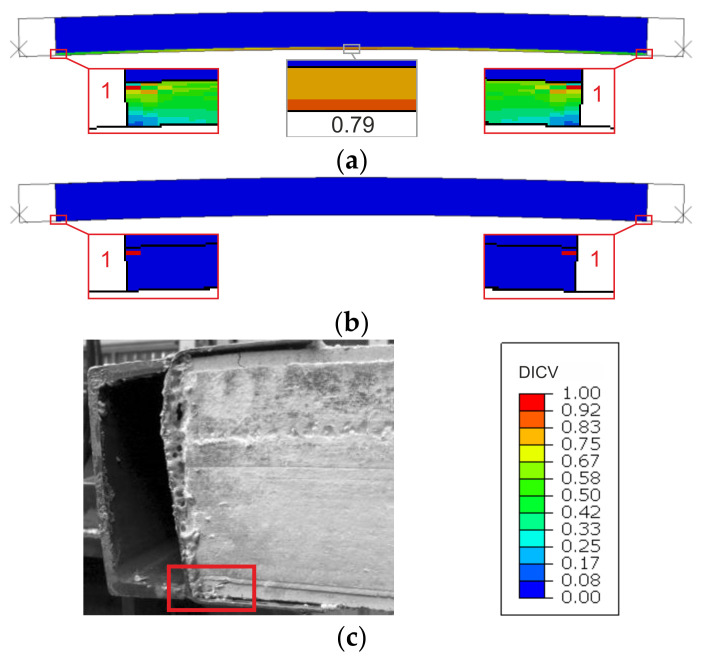
Comparison of failure modes in natural-scale e2 panel compression test; FEA results: (**a**) MgO min, (**b**) MgO max; (**c**) experimental observation.

A comparison between failure modes predicted in FEA and those observed in experimental tests is presented in Figure 14, Figure 15 and Figure 16. It can be seen that the DICV values in the MgO min variants are distributed more evenly across the facing subjected to stronger compression (Figure 14a, Figure 15a and Figure 16a), while the MgO max variants result in maps with distinct peak values concentrated on facing edges, in the contact zone with loading profiles (Figure 14b, Figure 15b and Figure 16b). In all cases, the DICV distribution maps indicate failure initiation on the edge of the facing subjected to higher intensity compressive stress for both facing material variants, which results in failure of the top facing in the *e* = 0 case, and failure of the bottom facing in two remaining cases. All predicted failure locations are in agreement with experimental observations (Figure 14c, Figure 15c and Figure 16c).

**Figure 17:**
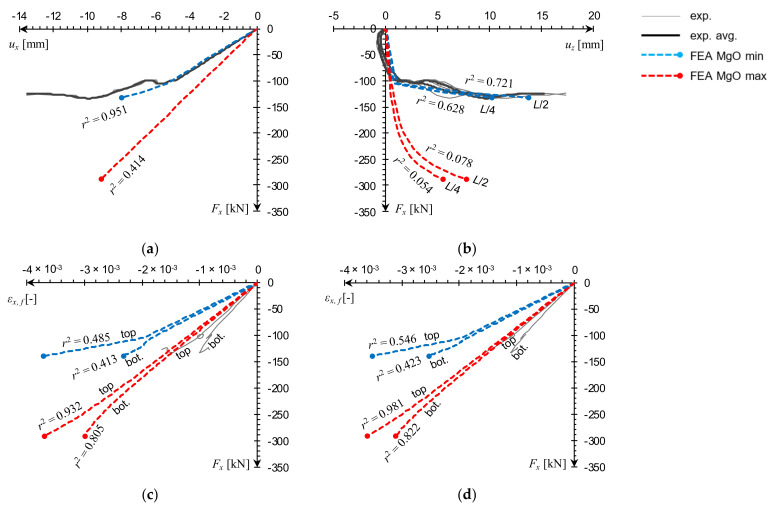
Comparison of natural-scale e0 panel compression test curves obtained from FEA against experimental data: (**a**) *F_x_*(*u_x_*), (**b**) *F_x_*(*u_z_*), (**c**) *F_x_*(*ε_x,f_*) at *L*/2, (**d**) *F_x_*(*ε_x,f_*) at *L*/4.

Data plots obtained from both experimental and numerical analyses were arranged into three categories: (1) force–displacement, *F_x_*(*u_x_*), (2) force–deflection, *F_x_*(*u_z_*), and (3) force–strain, *F_x_*(*ε_x,f_*). Experimental displacements were measured with LVDTs, and experimental strains were obtained as SG readings. Computational curves were compared with corresponding experimental curves (averaged, if available, individual, if not) by calculating the coefficient of determination, *r*^2^, for each pair of the resampled datasets [30]. The experimental test of uniaxial compression (*e* = 0) resulted in a failure load value *F_x_^u^* = 127 kN, which is unexpectedly low, as the corresponding results of both eccentric load tests were higher. However, the comparison with numerical outcomes showed that this result is actually within the FE model’s prediction range (Figure 17a,b). Numerical force–strain curves are very close to the experimental response as well (Figure 17c,d). It is worth to note an appearance of a small loop, clearly visible in all experimental force–strain curves around *F_x_* = 100 kN. A possible cause for this might be a material defect in one of the facings leading to localized damage, resulting in a premature drop of the ultimate load, and irregularities in *F_x_*(*ε_x,f_*) curves. This occurrence is in line with the results of previous research, which showed that compressive strength of the analyzed MgO board varies significantly from sample to sample [25,26].

**Figure 18:**
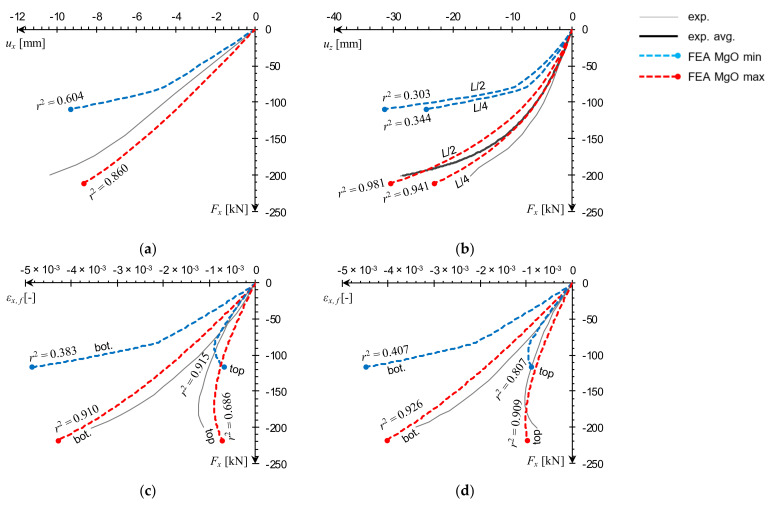
Comparison of natural-scale e1 panel compression test curves obtained from FEA against experimental data: (**a**) *F_x_*(*u_x_*), (**b**) *F_x_*(*u_z_*), (**c**) *F_x_*(*ε_x,f_*) at *L*/2, (**d**) *F_x_*(*ε_x,f_*) at *L*/4.

**Figure 19:**
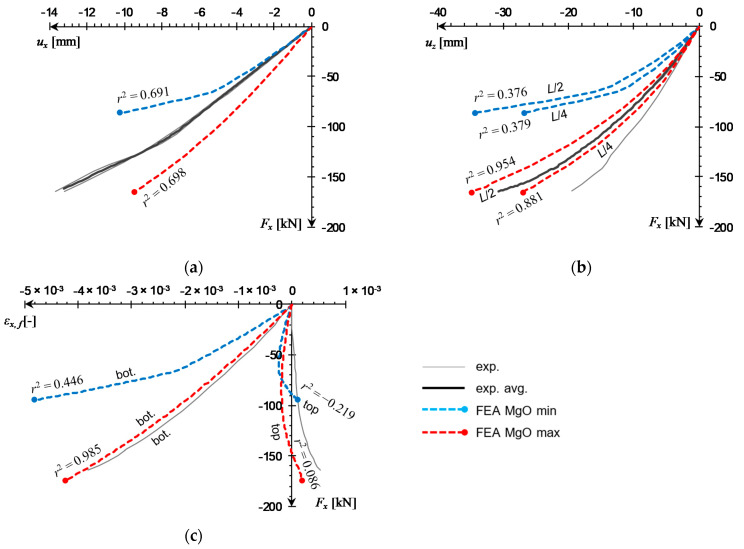
Comparison of natural-scale e2 panel compression test curves obtained from FEA against experimental data: (**a**) *F_x_*(*u_x_*), (**b**) *F_x_*(*u_z_*), (**c**) *F_x_*(*ε_x,f_*) at *L*/2.

The results of both eccentric load tests exhibit a similar level of agreement between numerical and experimental curves. The numerical force–displacement curves place themselves concentrically around the experimental data (Figure 18a and Figure 19a) and force–deflection curves in mid- and quarter-span are close to laboratory measurements for the MgO max outcomes (Figure 18b and Figure 19b). It can be seen that the FE model is able to reproduce the flexural deformation quite well, with deflection in *L*/2 being slightly more accurate than in *L*/4. The distinction between *L*/2 and *L*/4 deflections is quite apparent, unlike the uniaxial load case, in which the difference is barely visible (Figure 17b). Force–strain curves obtained from the MgO max variant are in very good agreement with experimental measurements in the e1 test (Figure 18c,d) and for the bottom facing in the e2 test (Figure 19c). The laboratory measurements at the top facing in the e2 test indicate strain being negative in the initial loading stage, and transitioning into tension for the remainder of the test. Both numerical curves remained mostly in the negative strain range and transformed into tension only near the end of the simulation. This qualitative difference led to very low *r*^2^ values, however, shapes of numerical curves still resemble experimental ones quite well.

A summary of FEA and experimental results is presented in Table 4. The experimental failure stress values were obtained from (4). The best overall numerical result accuracy was obtained for MgO min in the e0 panel and MgO max for e1 and e2 specimens.

## 4. Discussion

Experimental tests on samples of varying slenderness and load eccentricity values allowed to obtain a varied response for a comprehensive FE model validation. The low slenderness L1 (*λ* = 8.7) and L2 (*λ* = 20.4) samples failed by facing crushing without visible transverse deflection occurring before failure initiation. A significant increase of slenderness (*λ* = 60.4) in the L3 column caused a global buckling response accompanied by a pronounced flexural deformation leading to facing tensile failure. The full-scale panels’ slenderness (*λ* = 37.3) was slightly higher than L2 samples’ and no form of local or global buckling was observed. In the concentrically loaded e0 panel only a slight deflection caused by the gravitational force was noted; however, the introduction of load eccentricities in e1 (*e* = 27 mm) and e2 (*e* = 54 mm) panels resulted in pronounced transverse deflections. In the case of the e2 test, positive strain readings were recorded in the middle of the top facing; however, the bottom facing was subjected to intensified compression and the failure initiated on its edge.

The FE model was able to reproduce all of the effects listed above and allowed to reach a better understanding of processes taking place in CSIPs subjected to edgewise compression. The SSV maps, produced as results of the stress state identification, depicted changes taking place in all simulated samples in a physically sound way.

In the low-slenderness concentrically loaded numerical samples (L1, L2, e0) the SSV maps were dominated by values equal or close to −1 throughout the whole analysis. A slight deflection in the e0 specimen was interpreted by the algorithm as a minor shift towards shear in the core, whilst, the reminder of the deformable area was considered as under compression. Material parameter values used in these simulations were heavily centered around SSV = −1 dataset. Introduction of load eccentricity in e1 and e2 cases produced visible changes in SSV maps, signaling an increased variation of material parameter selection and reflecting the intensification of flexural action. The appearance of a region under tension in the upper facing of the e2 panel test simulation was captured on the SSV map as well. All of these simulations resulted in failure located on the facing edges, in complete agreement with laboratory test observations. Failure criteria used in FEA produced very similar ultimate stress values for all facing crushing cases, both small- and full-scale. This shows that compressive failure data obtained from small-scale laboratory tests can be used in a numerical analysis of full-scale CSIPs.

The simulation of the high-slenderness L3 column is the best showcase of the proposed model’s capabilities. At the initial stage, the SSV maps recognized the whole specimen as being under compression. When the reaction force reached a critical value both facings remained under compression, but a slight transverse deflection formed, accompanied by an SSV distribution shift towards shear in the core. The critical load values obtained from the model were in very good agreement both with experimental and analytical results. Further vertical displacement intensified the flexural deformation in the post-buckling range and caused a qualitative change in the SSV distribution: one facing remained under compression, substantial areas under tension appeared in the other facing, and most of the core was recognized as under shear. Throughout the whole analysis, material properties in different areas of the sample were assigned based on three different datasets corresponding to SSV = −1, SSV = 0, and SSV = 1. In the post-buckling range, the load-bearing capacity kept decreasing as the transverse deflection increased. At the final stage, failure initiation condition for the MgO board in tension was fulfilled first, resulting in a failure mode consistent with the experimental one.

The results showed that the quality of MgO board is a vital factor for computational accuracy, as it has a direct impact on how the facing material model is defined. The use of MgO min and MgO max descriptions was dictated by a substantial scatter in experimental results and it produced numerical results in form of ranges. It allowed to illustrate that even though the concentrically compressed e0 panel failed at lower load than both e1 and e2 specimens, it was actually within expectations based on small-scale MgO board strength study.

No local buckling or pre-failure delamination were observed in any of the experimental tests. Moreover, such behavior seems unlikely in the CSIP’s case, due to the brittle nature of the MgO board damage. Delamination was observed only after facing cracking occurred, and the sample lost its load-bearing capacity. This effect was not in the scope of the present study; however, perfect bonding between layers can be substituted with cohesive contact to track delamination progression if needed.

The presented results showed that the proposed model was able to identify all failure types correctly and capture effects characteristic to compression of various CSIP specimens. Consideration of material bimodularity with the use of author’s procedure allowed for accurate modeling of flexural action in case of high-slenderness and eccentrically loaded specimens. It is worth noting that even though the number of samples in each laboratory test was quite limited, the covered spectrum of geometries and loading conditions was wide enough to observe varied responses that were successfully reproduced in numerical simulations. Moreover, the proposed numerical approach was used with the same set of material parameter values to successfully reproduce CSIP failure under flexure [27], which further improves its reliability.

## 5. Conclusions

Numerical simulations of compression tests on CSIP specimens of varied slenderness, subjected to loads with different eccentricity values, were performed and compared with experimental data. The following conclusions can be drawn, based on the obtained results.

- The proposed stress state dependent numerical approach enables an automatic differentiation of elastic, plastic, and failure properties in the entire specimen throughout the whole analysis. This functionality allows accounting for flexural action caused by load eccentricity and global buckling. The presented SSV maps show that the procedure identifies stress state distribution changes in all CSIP samples in a physically sound manner.
- The numerical model identified all failure modes correctly. It was able to capture the e0 panel’s premature failure and global buckling of the L3 column. A high level of curve similarity for both force–displacement and force–strain curves was obtained as well. A few slight differences were noted that can be attributed to the idealization of boundary conditions in FEA.
- The model allows for efficient macroscale calculations and to avoid detailed mesoscale modeling. The author’s procedure enhances the capabilities of a homogenized approach in a straightforward manner.
- The availability of comprehensive material property information for different stress states is preferred; however, this approach allows for a simple introduction of additional data once it is obtained from experimental tests.

Based on the successful validation performed in this study, the FE model can be considered feasible for CSIP compression simulations. As the previous research [27] has proven it is suitable for flexural analysis, meaning that it can be applied to different loading conditions. Therefore, the proposed approach can be considered a reliable and robust aid for CSIP design. Moreover, the procedure can be supplemented to 3D problems where the core is discretized with continuum solid elements and the facings with structural shell elements. Further research in this direction is planned to test the approach with different kinds of CSIPs, SIPs, and other sandwich panels.

## Figures and Tables

**Table 1:** CSIP samples’ geometry and test setup parameters.

Sample	*n*	Core Type	*t_f_* mm	*t_c_* mm	*a* mm	*L* mm	*e* mm	Rotation at Supp.	*L_e_* mm	*λ*
L1	2	EPS15	11	20	100	275	0	Fixed	138	8.7
L2	2	EPS21	11	20	100	645	0	Fixed	323	20.4
L3	1	EPS15	11	20	100	955	0	Free	955	60.4
e0	1	EPS21	11	152	1000	2750	0	Free	3080	37.3
e1	1	EPS21	11	152	1000	2750	27	Free	3080	37.3
e2	1	EPS21	11	152	1000	2750	54	Free	3080	37.3

Note: *n* = number of tested samples; *t_f_*, *t_c_*, *a*, *L* = specimen dimensions (Figure 1); *e* = eccentricity; *L_e_* = effective length; *λ* = slenderness ratio.

**Table 2:** Material parameter values used in FEA [27].

Material Model	SSV	*E* MPa	*υ*	*σ_pl_* MPa	*E_pl_* MPa	*β*	*ψ*	*p_t_*_0_ MPa	*ε^pl,eq^*	*η*
MgO min	−1	2430	0.18	5.0	1205	25	10	8	1.6 × 10^−3^	−3.2 × 10^−1^
	1	6325	0.18	4.8	1940	25	10	8	1.4 × 10^−3^	3.3 × 10^−1^
MgO max	−1	3885	0.18	18.2	1130	25	10	8	3.0 × 10^−4^	−3.2 × 10^−1^
	1	8845	0.18	6.1	1495	25	10	8	1.3 × 10^−3^	3.3 × 10^−1^
EPS15	−1	5.0	0.09	0.075	0.14	1	1	0.7	1.0	−1.0
	0	6.1	0.09	0.075	3.45	1	1	0.7	8.3 × 10^−3^	−1.5 × 10^−2^
	1	7.2	0.09	0.135	4.08	1	1	0.7	8.0 × 10^−3^	3.3 × 10^−1^
EPS21	−1	6.8	0.12	0.090	0.18	2	2	0.5	1.0	−1.0
	0	9.2	0.12	0.090	5.21	2	2	0.5	1.4 × 10^−2^	−1.5 × 10^−2^
	1	10.5	0.12	0.160	5.94	2	2	0.5	7.1 × 10^−3^	3.3 × 10^−1^

Note: *E* = modulus of elasticity; *υ* = Poisson’s ratio; *σ_pl_* = yield stress; *E_pl_* = modulus of hardening; *β* = angle of friction; *ψ* = dilation angle; *p_t_*_0_ = initial hydrostatic tension strength; *ε^pl,eq^* = equivalent plastic fracture strain; *η* = stress triaxiality factor.

**Table 3:** Summary of small-scale FEA result similarity to experimental data.

Sample	Experimental		FEA			Comparison			Failure Mode Pred.
	*F_x_^u^* kN	*σ_x,f_^u^* MPa	Fac. Mat. Variant	*F_x_^u^* kN	*σ_x,f_^u^* MPa	*δF_x_^u^* %	*δσ_x,f_^u^* %	*r^2^*	
L1	−27.08	−14.13	MgO min	−18.93	−9.04	30.1	36.0	0.741	Correct
			MgO max	−40.51	−19.02	49.6	34.6	0.416	Correct
L2	−21.36	−13.71	MgO min	−18.91	−9.05	11.5	34.0	0.559	Correct
			MgO max	−40.49	−19.03	89.5	38.8	0.981	Correct
L3	−12.91 ^a^	−5.95 ^a^	MgO min	−7.48 ^a^	−3.47 ^a^	42.3	41.7	0.452	Correct
			MgO max	−9.83 ^a^	−4.98 ^a^	24.1	16.3	0.808	Correct

^a^ Buckling failure.

**Table 4:** Summary of full-scale test results.

Sample	Experimental		FEA			Comparison		*r*^2^ (-)							Failure Mode Pred.
	*F_x_^u^* kN	*σ_x,f_^u^* MPa	Fac. Mat. Variant	*F_x_^u^* kN	*σ_x,f_^u^* MPa	*δF_x_^u^* %	*δσ_x,f_^u^* %	*F_x_(u_x_)*	*F_x_(u_z_)* L/2	*F_x_(u_z_)* L/4	*F_x_(ε_x, f_)* L/2 top	*F(ε_x, f_)* L/2 bot	*F_x_(ε_x, f_)* L/4 top	*F_x_(ε_x, f_)* L/4 bot	
e0	−133.3	−5.77	MgO min	−133.0	−9.59	0.3	66.2	0.951	0.721	0.628	0.485	0.413	0.546	0.423	Correct
			MgO max	−288.0	−20.66	116.0	258.1	0.414	0.078	0.054	0.932	0.805	0.981	0.822	Correct
e1	−199.8	−12.35	MgO min	−109.1	−9.12	45.4	26.2	0.604	0.303	0.344	0.915	0.383	0.807	0.407	Correct
			MgO max	−211.2	−19.58	5.7	58.5	0.860	0.981	0.941	0.686	0.910	0.909	0.926	Correct
e2	−161.9	−10.10	MgO min	−86.3	−9.12	46.7	9.7	0.691	0.376	0.379	−0.219	0.446	-	-	Correct
			MgO max	−165.3	−19.59	2.1	94.0	0.698	0.954	0.881	0.086	0.985	-	-	Correct

## Data Availability

The data presented in this study are available on request from the corresponding author.
